# Structure and Strength of Bovine and Equine Amniotic Membrane

**DOI:** 10.3390/biology11081096

**Published:** 2022-07-23

**Authors:** Hannah C. Wells, Katie H. Sizeland, Nigel Kirby, Richard G. Haverkamp

**Affiliations:** 1 School of Food and Advanced Technology, Massey University, Palmerston North 4442, New Zealand; wells.hannah09@gmail.com; 2 ANSTO, Lucas Heights, NSW 2234, Australia; katie.sizeland@plantandfood.co.nz; 3 ANSTO, Clayton, VIC 3168, Australia; nigelk@ansto.gov.au

**Keywords:** amnion, collagen, SAXS, scaffold

## Abstract

**Simple Summary:**

Thin, strong scaffold materials are needed for surgical applications. There is a limited selection of available materials and new materials are required. Amnionic membrane from cattle and horses were investigated for this purpose. The structure of these materials was characterized with synchrotron techniques and the strength was measured. A possible relationship between the structure and strength was identified. These amnion materials from animal sources are strong, thin, and elastic materials, although weaker than some other collagen tissues. They may be suitable for use in surgery as an alternative to material from human donors.

**Abstract:**

Thin, strong scaffold materials are needed for surgical applications. New materials are required, particularly those readily available, such as from non-human sources. Bovine amniotic membrane (antepartum) and equine amniotic membrane (postpartum) were characterized with tear and tensile tests. The structural arrangement of the collagen fibrils was determined by small-angle X-ray scattering, scanning electron microscopy, and ultrasonic imaging. Bovine amnion had a thickness-normalized tear strength of 12.6 (3.8) N/mm, while equine amnion was 14.8 (5.3) N/mm. SAXS analysis of the collagen fibril arrangement yielded an orientation index of 0.587 (0.06) and 0.681 (0.05) for bovine and equine, respectively. This may indicate a relationship between more highly aligned collagen fibrils and greater strength, as seen in other materials. Amnion from bovine or equine sources are strong, thin, elastic materials, although weaker than other collagen tissue materials commonly used, that may find application in surgery as an alternative to material from human donors.

## 1. Introduction

Amnion is a thin collagen-based membrane that along with chorion makes up the innermost layer of the placenta, called the amniotic membrane or chorioamnion. This tissue consists of a layer containing trophoblast, mesenchymal cells, and collagen. The amnion, which is separated from the more cellular chorion layer at a well-defined interface, is dominantly acellular [1]. The role of the amniotic membrane is to encompass and support the developing fetus and surrounding amniotic fluid during pregnancy. In order to carry out this role, the membrane must be strong and resilient, yet flexible enough to accommodate for a growing fetus [2]. In addition, the amnion plays an important biological role in the development of a fetus, including the delivery of nutrients and excretion through diffusion.

Due to these features, human amnion has emerged as a useful biological material in a range of surgical applications. Recent reviews have covered the applications of amniotic membrane in surgery [3,4]. These reviews also cover the preparation of the tissue. These applications of amniotic membrane include for ophthalmic surgery, specifically corneal and scleral, which is the most widely reported use [5,6]. It has been applied for the treatment of non-healing wounds [7,8,9], bone regeneration [10], and peridontal surgery [11,12]. It has also been proposed for urology [13]. It is used in wound dressing and skin reconstruction [14,15,16], although other thicker scaffolds from acellular dermal matrix (ADM) are well-established in this application [17,18,19].

Currently, it is mainly human amniotic tissue that is used for medical applications. However, amnion from other animals has been investigated for use in a few cases, but so far only in veterinary applications. For example, bovine [20], equine [21], porcine [22], and canine [23,24] amniotic membrane material has been investigated for veterinary applications.

The strength of human amniotic membrane has been well-characterized. The tensile and burst strengths of chorioamnion have been measured as well as the separate chorion and amnion components. It has been found that about two thirds of the strength of the chorioamnion is in the amnion and one third in the chorion [25,26]. Fetal membrane physical properties (strength, stiffness) are very heterogeneous over the membrane surface [26]. A more useful measure of strength for medical surgical applications is crack propagation, which can be measured by burst strength [27]. The strength varies throughout gestation. Burst strength increases up to 20 weeks of gestation and then plateaus until 39 weeks of gestation, when it falls dramatically [28], so that the amnion is weaker after delivery [29]. This change is also reflected in the Young’s modulus [30].

Human amniotic transplantation, as with many human tissue materials, is limited by the availability of willing donors [31]. Therefore, as with other collagen-based materials for surgical applications, the use of heterografts could provide a large source of alternative material [20,21,22,23,24].

The purpose of this work is to characterize the structure and strength of non-human amniotic membrane from equine and bovine, as a possible biomaterial for surgical applications in humans. The strength and the structure of amniotic membrane from these sources is investigated and strength–structure relationships are considered.

## 2. Materials and Methods

### 2.1. Material

Equine and bovine amniotic membrane was sourced from AgriLabs (Waipukurau, New Zealand). Equine amnion was collected soon after birth. The bovine tissue was obtained from antepartum material from cows sent to slaughter at a recognized abattoir. The tissue was washed with water and stored whole at −60 °C. The tissue used in this work consists of the two layers, amnion and chorion, which we refer to as the amniotic membrane or chorioamniotic membrane in this work. Prior to sample analysis, it was thawed and cut into sample sizes. The amniotic membrane remained untreated for sample analysis unless otherwise specified.

### 2.2. SAXS Measurement

The SAXS/WAXS beam line at the Australian Synchrotron was used to collect diffraction patterns. A high-intensity undulator source with an X-ray energy of 12 keV was used. An energy resolution of ~10^−4^ from a cyro-cooled Si (111) double-crystal monochromator was used with a beam size of 250 × 80 µm FWHM focused on the sample and a total photon flux of about 2 × 10^12^ phs^−1^. A Pilatus 1 M detector with an active area of 170 × 170 mm was used with a sample-to-detector distance of 3371 mm. Equine and bovine amnion samples were mounted both flat and edge-on to the incoming X-rays. For edge-on samples, diffraction patters were recorded every 0.1 mm through the cross-section of the samples.

Scatterbrain software was used for the initial SAXS data analysis. Then d-spacing of the collagen fibrils was determined from integrated intensity plots of the azimuthal range of 45° to 135° by fitting a Gaussian curve to the sixth-order diffraction peak and determining the center position of the curve.

The orientation index (*OI*) is defined by:

$$OI=\frac{90{}^{\circ}-OA}{90{}^{\circ}}$$

where *OA* = orientation angle, the minimum azimuthal angle range centered around 180° that contains at least 50% of the collagen fibrils based on the method of Sacks for light scattering [32], but converted to an index using the spread in azimuthal angle of d-spacing diffraction peaks. *OI* provides a measure of the spread of collagen fibril orientation, where an *OI* of 1 indicates that the fibrils lie parallel to each other and an *OI* of 0 indicates isotropic orientation. *OI* is essentially equivalent to Herman’s orientation factor.

Data were analyzed using Scatterbrain software [33] for initial processing then custom software for analysis of *OI*.

### 2.3. Differential Interference Contrast Microscopy (DIC)

DIC images were recorded on an Olympus IX83 inverted microscope.

### 2.4. Atomic Force Microscopy (AFM)

Square samples were cut from the untreated bovine amnion and mounted onto a magnetic metal disc 12 mm in diameter using double-sided tape with the inner (fetal) surface imaged. An Asylum Research MFP-3D atomic force microscope was used. NSG01 cantilevers (NT-MDT, Moscow, Russia), which had a force constant of about 5 Nm^−1^, were used for imaging in AC mode.

### 2.5. Ultrasonics

Ultrasonic images were recorded with a DermaScan C USB instrument (Cortex Technologies, Hadsund, Denmark). A 50 MHz 2D-scanning head was used to carry out scanning at 6–8 frames per second over a distance of 12.1 mm in 224 steps. The scanning head contains an internal water chamber to minimize attenuation of the acoustic signal. The sound velocity in amnion was not calibrated but was assumed to be 1580 m/s, the standard value for skin. The bandwidth was 0.75, which resulted in an axial resolution of 30 µm. The lateral resolution was around 60 µm. The transducer gain level and gain profile were used to adjust the amplification of the signal. Contact between the window of the ultrasound probe and the sample was facilitated by Vue ultrasound gel (Optimum Medical Solutions, Leeds, UK). One difficulty with thin-sample imaging can be distinguishing the boundary between the sample and the support (in this case a polypropylene sheet). An effective way to solve this was found using the ultrasound gel between the amnion and the support, resulting in a non-reflective band below the amnion.

The ultrasonic data can be displayed in what is conventionally called an A-scan or a B-scan. An A-scan is a line scan that represents depth information from one point on the surface of the leather, while a B-Scan is a two-dimensional image that represents a cross-sectional area of the amnion (and is composed of a large number of A-scans but displayed using color for intensity). We present the data as B-scans.

### 2.6. Tear and Tensile Test

Tear tests were carried out in accordance with ISO 3377:2016-2 for double-edge tear testing using a TA.XTplus Texture Analyzer (Stable Micro Systems Ltd., Godalming, UK) and a slot tear rig. Samples were thawed from frozen (−60°) and cut using a press knife just before testing. Sample thickness was measured and recorded using a digital micrometer.

Tensile tests were also carried out on the TA.XTplus Texture Analyzer using the self-tightening roller grips to prevent the material from slipping. The tests were carried out in accordance with standard ISO 3376:2011. Samples were thawed from frozen and cut using a press knife into the dog-bone shape ready for tensile analysis. Sample thickness was measured using a digital micrometer. Stress–strain curves were recorded by carrying out uniaxial stretching with the sample mounted vertically. The sample was strained at a rate of 2 mm s^−1^.

Thickness for the tear and tensile test samples was measured using an Insize 2364-10A thickness gauge (Insize, Loganville, GA, USA) with two 10 mm-diameter pads which apply a pressure of around 1.2 kPa to the sample.

Two animals each of bovine and equine were tested, with 17 samples of bovine and 14 samples of equine.

Sigmaplot was used for the statistical analysis.

## 3. Results

### 3.1. Membrane Material

**Figure 1:**
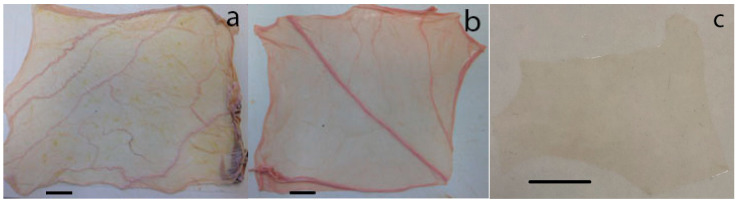
(**a**) Bovine chorioamniotic membrane, (**b**) equine chorioamniotic membrane, (**c**) bovine amnion (separated from the chorioamnion). Scale bar: 25 mm.

The chorioamniotic membranes have a heterogeneous structure (Figure 1a,b) which creates difficulties in obtaining representative portions for tear and tensile testing for structure analysis. However, the amnion (Figure 1c) appears much more homogenous and therefore suitable for preparing materials for surgical applications.

### 3.2. Differential Interference Contrast Microscopy

**Figure 2:**
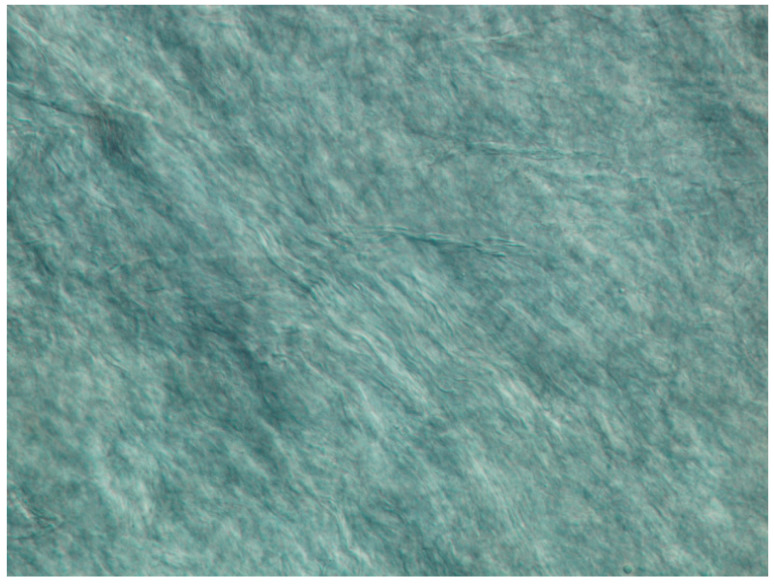
Differential interference contrast (DIC) microscopy image of the surface of the bovine amnion. Image width is 300 µm (magnification ×310).

The DIC image of the surface of the bovine amnion clearly shows the collagen fibril bundles (Figure 2). There is some alignment apparent in these fibril bundles, and a degree of crimp (the wavy shape of the fibril bundles).

### 3.3. Ultrasonics

**Figure 3:**
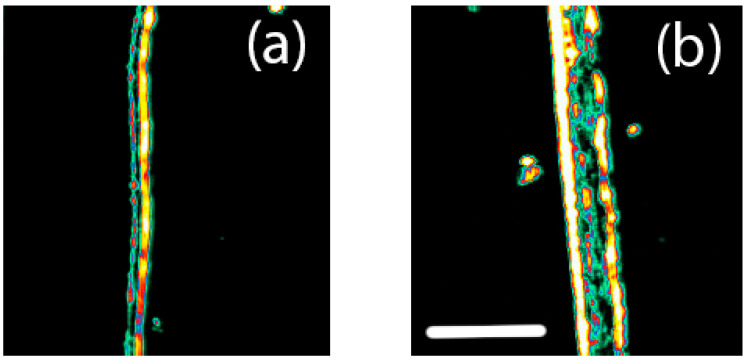
Ultrasonic B-scans of (**a**) bovine amnion and (**b**) equine chorioamnion (amnion on the left of the image, chorion on the right). Scale bar: 500 µm. The sensor measured from the lefthand side of the image.

The ultrasonic B-scans of the amnion show the layered structure (Figure 3). The chorion and amnion can be seen as separate layers in the ultrasonic images of the chorioamniotic membrane. The thickness from this imaging for the bovine amnion is around 95 μm and 220 μm for the equine chorioamniotic membrane in this hydrated state.

### 3.4. Atomic Force Microscopy

**Figure 4:**
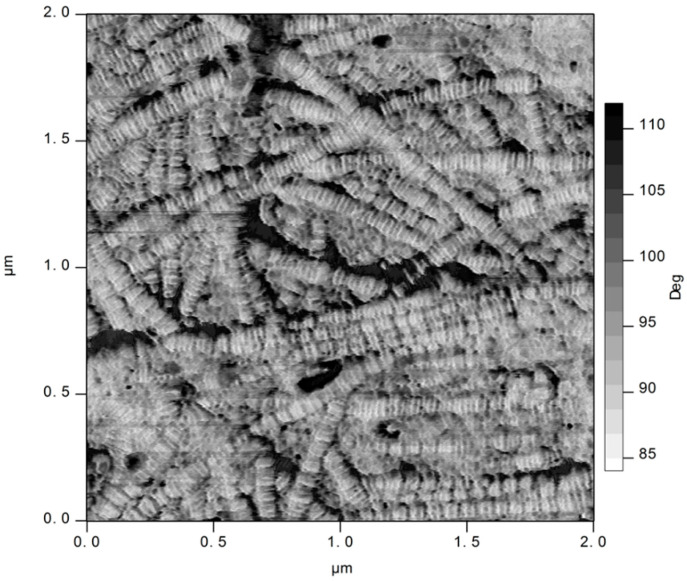
AFM image of the bovine amnion taken on the inner (fetal) surface. A phase image, representing the shift in phase of the oscillating AFM cantilever as it interacts with the sample (magnification ×37,000).

A dense network of collagen fibrils was visible on the inner (fetal) surface of the bovine amnion with AFM (Figure 4). The banded structure of type I collagen can be clearly seen. The fibrils form a network, with some alignment.

### 3.5. Tear Strength

The tear strength of the equine amniotic membrane is similar to but slightly larger than that of the bovine amniotic membrane (Table 1). The difference was slightly larger for the thickness-normalized tear strength, which yields a comparative measure of the properties of the material making up the membrane.

A *t*-test for the difference in thickness-normalized tear strength between equine and bovine found that the apparent difference was not statistically significant and may just be due to chance.

### 3.6. Tensile Strength

The tensile strength of the equine amniotic membrane is similar to but slightly smaller than that of the bovine amniotic membrane (Table 2). This is the opposite relationship to the tear strength. The difference was maintained in the thickness-normalized tear strength.

### 3.7. SAXS of Collagen Structure

**Figure 5:**
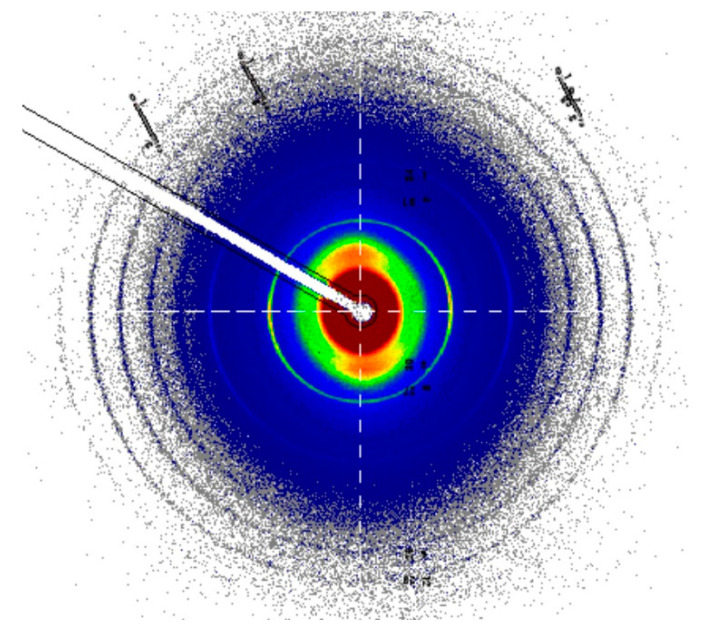
Representative SAXS pattern of the equine amniotic membrane.

The SAXS patterns of the chorioamniotic membrane show the banded structure of fibrillar collagen, the rings visible in this pattern, with some orientation of the fibrils apparent, most easily seen near the central part of the pattern (Figure 5). The d-spacing of the fresh tissue (wet) was similar in equine and bovine amnion (Table 3). A moderate amount of orientation of the fibrils, quantified as the orientation index (OI), was present in both the amniotic membrane materials (Table 3).

## 4. Discussion

### 4.1. Strength Compared with Human Amnion and Other Materials

Amniotic membranes from equine and bovine sources have reasonable tensile strengths of 2.5 and 2.8 MPa, respectively. This is higher than the human amniotic membrane, although the published reports on human amnion are wide-ranging. One review of a large number of studies of human amnion presented a mean tensile failure stress of 0.9 MPa from range of values (0.3–1.8 MPa) [34]. The strength is distributed in chorioamniotic tissues, with about one third of the strength in the chorion and two thirds in the amnion [25,26]. The only study we found of amnion from other than human tissue recorded a tensile strength of 0.11 (0.10) MPa for canine amnion [24], lower than for human amnion.

The bovine amniotic membrane studied here was antepartum, while the equine amniotic membrane was postpartum. It has been reported that there are small differences in the Young’s modulus [30] and tensile strength of human amnion antepartum and postpartum, with the tensile strength of the chorioamniotic membrane reducing from 1.86 to 1.04 MPa before and after delivery [29], although other work has suggested that the differences are small [35]. The human amnion used as a surgical material is normally obtained from postpartum tissue. This preliminary work on bovine and equine amniotic membrane does not investigate any differences in strength or structure between antepartum and postpartum tissue.

While tensile strength is commonly measured, it has been shown in other materials that tear strength is a much better measure of performance for materials in application. Tear strength can be measured in a number of ways, including a tear test, a burst test, or a suture pullout test. These tests are similar in that the failure mode depends on tearing from a point of stress. However, it is not possible to directly compare the different tests. Therefore, while data have been published for burst strength in human amnion [27,28], we cannot numerically compare these values with the work presented here.

We can, however, make a comparison between the tear strength of the amniotic membrane measured here with a range of other materials, both natural and treated, that are used in surgical applications (Table 4). Amniotic membrane does not compare favorably in strength with these other materials, when compared on a thickness-normalized basis. However, amniotic membrane is a very thin material, so where a thin scaffold is required, this property is prioritized over the lower tear strength of this material.

### 4.2. Structural Arrangement

The thickness-normalized strength of tissues is related to the structure of the material. The differences between different tissues and amniotic membrane from different sources must be due to differences in structure. One structural aspect, that we have been able to study here, is the arrangement of the collagen fibrils in these tissues. Two of the features of collagen fibrils and fibers in tissue are crimp (the wavy nature of the fibers) and the amount of orientation or alignment of the fibrils (quantified as the orientation index).

The layers of chorion and amnion are clearly seen as separate in the ultrasonic imaging, with a region that is less reflective to ultrasound between the layers, consisting of fluid rather than tissue.

Crimp is an important property found in tissues that are highly elastic [38,39,40] but is less prevalent in dermis. Amnion appears to have a moderate amount of crimp, visible at the scale of the optical images (Figure 2). This structure is due to fibrillar collagen, which is clearly dominant, as seen in the AFM image of the surface of the amnion (Figure 4). The crimp in amnion is more than in dermis but perhaps not as much as in pericardium. Crimp is associated with a highly elastic phase under low tensile forces, so this is commensurate with the requirements of amnion as the container for the fetus, somewhere intermediate between that of pericardium to accommodate the regular beating of the heart and that of skin, where flexibility is required but not large extensions.

The SAXS analysis showed that the collagen fibrils in the amniotic membrane material are more aligned than in pericardium and dermal materials (Table 4). Fibril alignment has been shown to have a strong relationship with strength, with more aligned fibrils leading to stronger material in dermis [18,41], pericardium [36,37,42], and even bone [39]. It is surprising then that the amniotic membrane has a lower normalized tear strength despite having higher fibril alignment (higher OI) relative to other tissues. However, this may be due to the amniotic membrane being a very variable material [26], which makes measurements of the mechanical properties fraught because small defects in a portion of material being studied can result in premature failure by aiding crack initiation or propagation [27]. Collagen tissues in general are very good at deflecting stress away from point sources [43], but the major “defects” that occur in the amniotic membrane may be greater than the ability of the tissue to respond.

Other factors play a part in the strength of tissues, including crosslinking glycosaminoglycans and other components such as elastin. Glycosaminoglycans are generally considered to crosslink collagen and provide strength and stiffening [44,45], although there may be some evidence against this [38]. Elastin is particularly important for the mechanical properties at small stress values and small tissue extension, assisting the recoiling mechanism [46]. These influences were not measured here.

There may be variations in tissue strength from different regions of the amnion, and this was not specifically measured in this study.

However, despite the amniotic membrane having a lower material strength than some other commonly used matrix materials, it has the advantage of being a very thin tissue compared with dermal materials so it is useful in applications that require such thin materials.

### 4.3. Application

Bovine and equine amniotic membranes have been shown to have good strength that could make these materials suitable as heterografts to replace human amniotic tissue in a variety of surgical applications. These tissues would need to be decellularized prior to implantation to avoid rejection. Other heterograft-decellularized materials are widely used [18]. This could help to alleviate the pressure of obtaining materials from human donors. The higher strength of the bovine and equine materials may be an advantage in applications requiring high strength.

## 5. Conclusions

Bovine (antepartum) and equine (postpartum) amniotic membranes were characterized with tear and tensile tests. These materials were shown to have higher tensile strength than the human amniotic membrane reported in previous literature. The tear strength was similar in the bovine and equine materials. Based on the thickness-normalized tear strength, a materials property, the amniotic membrane does not compare favorably with other commonly used surgical scaffold materials, when compared on a thickness-normalized basis. However, the amniotic membrane is a very thin material, so where a thin scaffold is required, this property might be prioritized over the lower tear strength of this material. The structure of the amniotic membrane was investigated. The tissue contains collagen fibers that are crimped, providing elasticity. The collagen fibrils are moderately aligned edge-on to the tissue, a feature that correlates positively with strength. However, this alignment is about double that of the stronger dermal and pericardial scaffold materials, which would suggest that the amniotic membrane should be stronger, and this may be due to the heterogeneous nature of the amniotic membrane enabling crack initiation and propagation. Amniotic membranes from bovine or equine sources are strong, thin, elastic materials that may find application in surgery as an alternative to amniotic membranes from human donors.

## Figures and Tables

**Table 1:** Tear strength of chorioamnion.

Amnion Type	Average Max Tear Force (N)	Average Material Thickness (mm)	Thickness-Normalized Tear Strength (N/mm)
Equine	2.95 (1.03)	0.20 (0.06)	14.8 (5.3)
Bovine	2.685 (0.92)	0.21 (0.03)	12.6 (3.8)

**Table 2:** Tensile strength of chorioamnion.

Amnionic Membrane Type	Average Max. Tensile Force (N)	Average Material Thickness (mm)	Thickness-Normalized Tensile Strength (MPa)
Equine	4.46 (1.73)	0.20 (0.07)	2.46 (1.23)
Bovine	5.85 (0.42)	0.21 (0.03)	2.79 (2.05)

**Table 3:** Collagen fibril structural parameters from SAXS.

Amnion Type	Average Collagen OI (σ)	Average Collagen d-Spacing (nm) (σ)
Equine	0.681 (0.05)	64.7 (0.25)
Bovine	0.587 (0.06)	64.5 (0.25)

**Table 4:** Structure and strength comparison of acellular dermal matrix, pericardium, and amniotic membrane.

Material Type	Species	Average OI Edge on	Average d-Spacing	Thickness of Material (mm)	Thickness-Normalized Tear Strength (N/mm)
Surgical scaffolds (ADM)	Human ADM [18]	0.23	64.60	1.06	79.0
	Porcine ADM [18]	0.40	64.20	1.63	43.2
	Bovine Neonatal ADM [18]	0.34	64.08	2.69	90.2
	Bovine Fetal ADM [18]	0.43	64.00	0.98	78.0
Native Pericardium	Bovine adult [36]	0.58–0.67		0.36	
	Bovine neonatal [36]	0.76–0.80		0.12	
	Bovine adult [37]	0.47			
	Bovine neonatal [37]	0.61			
Native amniotic membrane	Bovine amniotic membrane	0.59	64.56	0.21	12.6
(This work)	Equine amniotic membrane	0.68	64.70	0.20	14.8

## Data Availability

Data are available from the authors upon request.
